# Evaluation of a Bi-Analyte Immunoblot as a Useful Tool for Diagnosing Dermatitis Herpetiformis

**DOI:** 10.3390/diagnostics11081414

**Published:** 2021-08-05

**Authors:** Justyna Gornowicz-Porowska, Agnieszka Seraszek-Jaros, Magdalena Jałowska, Monika Bowszyc-Dmochowska, Elżbieta Kaczmarek, Marian Dmochowski

**Affiliations:** 1 Department and Division of Practical Cosmetology and Skin Diseases Prophylaxis, Poznan University of Medical Sciences, 33 Mazowiecka Street, 60-623 Poznań, Poland; 2 Autoimmune Blistering Dermatoses Section, Department of Dermatology, Poznan University of Medical Sciences, 49 Przybyszewskiego Street, 60-355 Poznań, Poland; mjalowska@ump.edu.pl (M.J.); mkdmoch@wp.pl (M.D.); 3 Department of Bioinformatics and Computational Biology, Poznan University of Medical Sciences, 4 Rokietnicka Street, 60-806 Poznań, Poland; agnetpa@gmail.com (A.S.-J.); elka@ump.edu.pl (E.K.); 4 Cutaneous Histopathology and Immunopathology Section, Department of Dermatology, Poznan University of Medical Sciences, 49 Przybyszewskiego Street, 60-355 Poznań, Poland; m.bowdmo@wp.pl

**Keywords:** dermatitis herpetiformis, diagnosis, tissue transglutaminase, gliadin

## Abstract

Immune responses to tissue transglutaminase (tTG) and nonapeptides of gliadin (npG) are associated with dermatitis herpetiformis (DH), a gluten-related dermatosis. Recently, a bi-analyte immunoblot (b-aIB) was introduced to detect IgA antibodies in response to tTG and npG. We compared the utility of ELISA and b-aIB with tTG in serological diagnoses of DH and their agreement with direct immunofluorescence (DIF). In total, 55 sera (27 DIF-positive DH patients, 4 DIF-negative DH patients and 24 healthy controls) were examined. ELISA for anti-tTG IgA, b-aIB for anti-npG and anti-tTG IgA, and statistical analysis were performed. The b-aIB with tTG showed 78% sensitivity, 100% specificity, 100% positive predictive value, and 82% negative predictive value in relation to ELISA. A better rate of agreement (Cohen’s kappa values) in IgA detection was observed in the pair tTG ELISA and b-aIB with npG (0.85) than in pairs tTG ELISA and b-aIB with tTG (0.78) or b-aIB with tTG and b-aIB with npG (0.78). No degree of agreement was found between serological tests and DIF. Both serological tests may be used to detect the anti-tTG IgA in DH patients. Still, DH diagnosing requires careful consideration of clinical data as well as results of tissue imaging (crucial DIF) and immunoserological techniques detecting DH-type features.

## 1. Introduction

In humans, nine members of the transglutaminase (TG) family have been identified [1,2], most of which catalyze post-translational protein-modifying reactions and thus, are able to alter their function [2,3]. The TG family is probably implicated in many diseases, such as type 2 diabetes, essential hypertension, neurodegenerative diseases, and dermatological disorders [3,4,5]. Some data have indicated anti-TG6 IgA in sera from schizophrenia patients [6].

TG1, TG3, and TG5 are important in the formation of stratum corneum in the skin and thus affect the integrity and function of the epidermis [7]. Autoimmunity to TG2, TG3, and TG6 may be associated with gluten intolerance and manifest as coeliac disease (CD), dermatitis herpetiformis (DH), or gluten-dependent neurological symptoms [2].

DH is a chronic IgA-mediated blistering dermatosis related to dietary gluten, where both autoimmunity and autoinflammation are implicated in its development [8,9,10,11,12]. The clinical picture presents an intense itching and polymorphic eruption, with a predilection for the external surfaces of the knees, elbows, buttocks, and shoulders, undergoing the spatial-temporal evolution.

DH is associated with gluten intolerance [10,11,12]; however, its linkage with CD still remains a matter of debate. Both diseases share the same HLA haplotypes (DQ2 and DQ8) [13]. An inflammatory infiltrate in DH is composed mainly of neutrophils that can be activated by diverse stimuli [14].

Direct immunofluorescence (DIF), demonstrating IgA deposits in the dermal papillae or/and along the dermal-epidermal junction, is the most important/prime criterion for a DH diagnosis [15]. However, some data postulate that DIF is a costly assay, primarily due to the large number of antibodies required [16]. Thus, Bresler et al. [16] argued that, although highly sensitive, DIF is not a standalone test for the diagnosis of DH.

Epidermal transglutaminase (TG3, eTG) and closely related tissue transglutaminase (TG2, tTG) are considered to be autoantigens in DH [8,9,10,17,18]. Anti-tTG IgA antibodies are also diagnostic markers for enteropathy in DH patients [18,19,20]. A previous report [21] suggested that circulating anti-tTG IgA may differentiate DH patients from those with linear IgA blistering dermatoses. Moreover, the levels of IgA anti-tTG antibodies reflect the extent of histopathologic changes of the jejunal mucosa in DH [22]. As was postulated by Salmi et al. [22], ELISA-based IgA-class tTG antibody tests should be the first-line serological test used when DH or CD is suspected.

The diagnosis of DH involves a tissue examination where DIF is the golden standard as well as the most specific diagnostic tool for DH and complementary serological analysis. The immunoserological diagnosis of DH usually involves the detection of IgA antibodies directed against transglutaminases (eTG, tTG). IgA antibodies to nonapeptides of gliadin (npG) evaluation and haplotyping have been suggested for diagnosing DH [23].

Although eTG is considered the main autoantigen in the cutaneous pathology of DH, analysis of diagnostic accuracy of different monoanalyte ELISA tests (eTG, tTG, npG IgA ELISAs) in our previous work [10] indicated anti-tTG IgA as the best choice for serological immunodiagnosis of DH. However, a definite correlation between TG function or deficiency and a specific pathology in DH still remains unclear.

Nowadays, a multiplex approach for diagnosing autoimmune blistering dermatoses [24,25], including multianalyte indirect immunofluorescence (IIF), multivariant profile ELISA, and strips of immunoblot assay bearing immobilized antigens, is developed. It seems that in the near future, traditional monoparametric ELISA diagnostics will be replaced by multianalyte/multiplex ELISA strategies [26].

It is postulated that IgA Fc receptors, including CD89, in DH may be related to neutrophil activation, production of autoantibodies, and gluten transport and/or transformation [27,28,29].

According to the recent literature data, several new recommendations have been reported about the clinical and immunopathological traits of DH [30]. Therefore, an update on the diagnosis of DH is needed. In light of this, a new bi-analyte immunoblot test (b-aIB) detecting IgA against npG and tTG in a simultaneous way was developed and introduced.

Diagnosing DH is not an easy task [31,32] and requires differentiation across the spectra of blistering autoimmune diseases and wheat/gluten-related disorders, as well as from any chronic itchy rash. Therefore, the aim of this study was to compare the diagnostic accuracy of the b-aIB and monoanalyte ELISA test, detecting anti-tTG IgA in serological diagnostics of DH, and to examine the diagnostic value/agreement of the b-aIB in comparison with traditional DIF in Polish DH patients.

## 2. Materials and Methods

This work was approved by the local Polish Ethical Committee of the Poznan University of Medical Sciences (no 1104/18).

### 2.1. Patients and Serum Samples

In total, 55 Slavic individuals were evaluated. Serum samples were obtained from patients with DH (31 individuals: 14 men and 17 women) as well as from healthy individuals (negative control, 24 donors). DH sera were deliberately selected to avoid the possibility of DH sex bias influencing the results. Sera were investigated to assess the diagnostic agreement between the b-aIB and monoanalyte ELISA in relation to the traditional diagnostic strategy (DIF). All DH serum samples were examined in the autoimmune blistering dermatoses section and cutaneous histopathology and immunopathology section in the Department of Dermatology, Poznan University of Medical Sciences, Poland. All healthy controls were not relatives of DH patients and gave no history of intolerance to gluten.

The peripheral blood used in the serological tests was obtained at the time of hospital admission/ambulatory care. The samples were centrifuged for 10 min at 3500 rpm. Thereafter, they were stored at −20 °C until performing ELISAs and b-aIB. Skin tissues were frozen and then subjected to a 4 μm sectioning, followed by mounting on poly-L-lysine coated glass slides.

Patients in the examined group of DH had to have clinical features (active pruritic, polymorphic skin rash suggesting DH) and at least one positive laboratory test (out of two). Laboratory tests included (i) cutaneous IgA deposition in any of seven possible diagnostic patterns seen with conventional DIF [15] and corroborated in certain by histological picture with hematoxylin and eosin (H&E) staining; (ii) DH-compatible IgA immune response at the molecular-biochemical serological level.

Our purposeful selection of DH patients included DIF-positive individuals (27 cases) and DIF-negative (4 cases) individuals. The diagnoses of DH were made whenever patients met criteria put forward by Beutner et al. [33] with some modifications [15].

**Figure 1:**
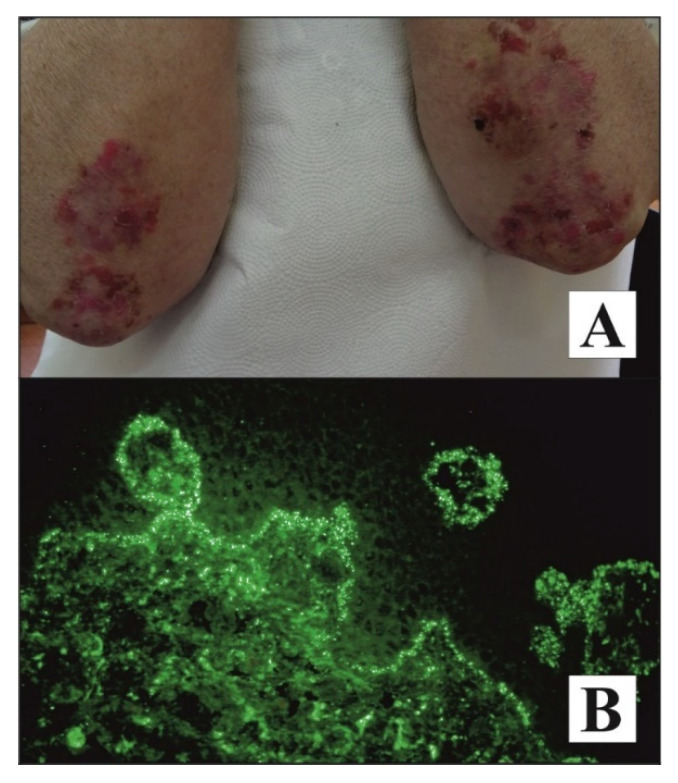
Noteworthy patients with DH. A pruritic, symmetrical rash composed of evolutionary lesions or blisters on an erythematous base located mainly on 1/3 proximal extensor surface of forearms in an elderly woman, having a daughter with mutilating rheumatoid arthritis, with DH developed after vaccination against influenza who showed an elevated level of anti-tTG IgA with ELISA and positive result of b-aIB for anti-npG IgA (**A**). Microgranular IgA deposits (++) at the tips of dermal papillae and along the dermal-epidermal junction revealed using direct immunofluorescence of perilesional skin visualized with short arc mercury lamp-operated microscopy in a middle-aged man with both DH and type 1 diabetes (DIF, original objective magnification ×40) (**B**).

Noteworthy patients with DH are presented in Figure 1.

Demographic data and detailed characteristics of patients participating in the study are presented in Table 1.

### 2.2. ELISA

The levels of serum IgA autoantibodies against tTG were assessed with Anti-tTG ELISA (Euroimmun, Lübeck, Germany), with the manufacturer’s cutoff value of 20 RU/mL, recommended by the producer as useful in DH diagnosis. All measurements were made using a programmable ELISA reader with MikroWin 2000 software.

### 2.3. Bi-Analyte Immunoblot Analysis

To analyze target antigens, sera were tested with the Euroline Coeliac Disease Profile (IgA) (Euroimmun, Lübeck, Germany), allowing parallel detection of anti-gliadin (GAF-3X, npG) and anti-tTG IgA. This test is a membrane strip with a combination of recombinant tTG and recombinant gliadin-analog fusion peptide separately. After blot strip blocking, sera were incubated at 1/100 for 30 min at room temperature. To detect the bound antibodies, a second incubation was carried out using alkaline phosphatase-labeled anti-human IgA. For the interpretation, a EUROLine Scan software (Euroimmun) was used.

### 2.4. Direct Immunofluorescence and Microscopic Examination

DIF of perilesional skin was performed in all cases for the detection of IgA, IgM, IgG, and C3 deposits. The tissue sections were incubated in a humid chamber for 30 min at room temperature (RT) with commercially available fluorescein isothiocyanate (FITC)-conjugated anti-human IgA, IgM, IgG, and C3 rabbit polyclonal antibodies (Dako, Denmark). The antibodies were used at a working dilution of 1:100 in phosphate buffer saline (PBS). The samples were then washed in PBS (pH 7.2) at RT for 15 min with gentle agitation. Then, slides were coverslipped and examined. Skin samples were examined by up to three independent observers with different methods, including blue light-emitting diode technology-operated microscopy (EuroStar III Plus microscope, Euroimmun, Germany) and short arc mercury lamp-operated microscopy (BX40, Olympus, Tokyo, Japan).

The intensities of deposits on slides were reported according to the arbitrarily assigned semiquantitative four-point scale (from “−” to “+++”) at identical objective magnifications (×20; ×40).

### 2.5. Statistical Analysis

Statistical analyses were performed using the statistical analysis software Statistica PL 13.0 (StatSoft, Inc, Tulsa, OK, USA).

The accuracy of the b-aIB was evaluated by calculating diagnostic sensitivity, specificity, reliability, as well as positive and negative predictive values in relation to monoanalyte ELISA using the dedicated MedCalc Software 2015 (Ostend, Belgium; www.medcalc.org; license valid until 8 September 2021; version 19.8). Estimates of sensitivity and specificity were calculated by tabulating the number of correctly classified samples.

Associations in the results between tests were assessed using Fisher’s exact test.

Cohen’s kappa was used to evaluate the interrater analytical agreements among these two systems for each of the antibodies tested and the DIF.

A *p* < 0.05 was considered statistically significant.

## 3. Results

The detailed results in the examined subgroup of DH patients with DH-compatible clinical features and positive DIF, as well as in the examined subgroup of DH patients with DH-compatible clinical features, but negative DIFs were presented in Table 2. In the control group (healthy subjects), there were no positive results of anti-tTG IgA with ELISA.

The diagnostic sensitivity and specificity, as well as positive and negative predictive values of b-aIB, in comparison with standard ELISA, are shown in Table 3.

The interrater agreements (Cohen’s kappa values) among methods are presented in Table 4 and Table 5.

There was an association between the positivity/negativity of results obtained with tTG b-aIB, npG b-aIB, and ELISA (*p* < 0.05). There was an association between the positivity/negativity of results obtained with tTG b-aIB and npG b-aIB (*p* < 0.05). There was no association between anti-tTG and npG IgA detection in DH patients (b-aIB, ELISA) and DIF (*p* = ≥ 0.05).

## 4. Discussion

The number of undiagnosed cases of DH, similar to CD [34], seems to be high. This may be partially the result of incorrect diagnoses and/or a diagnostic delay [35]. Thus, a single simple serological test facilitating DH recognition is desirable [36]. In light of this, anti-TG antibodies seem to play an important role in the histopathogenesis of DH [37,38,39,40,41], and the presence of circulating anti-tTG is often used to aid in the diagnosis and follow-up of these patients. However, it should be noted that possible immune reactions between tTG and eTG may lead to diagnostic pitfalls. There are two types of anti-eTG antibodies documented: (i) that bind to eTG exclusively, or (ii) that cross-react with tTG, which is in part due to high structural homology between the tTG and eTG molecules within its enzymatically active domains [11,37,39]. Moreover, the phenomenon of epitope spreading from tTG to eTG could determine IgA anti-eTG autoantibody production in a subset of coeliac patients who then develop DH [41].

In this study, we compared two immunoserological assays (b-aIB analysis and ELISA system) in terms of their use to detect anti-tTG IgA in the diagnosis of DH in a defined Polish population. To date, to the best of our knowledge, no previous study investigated the usefulness of the b-aIB in the DH diagnosing process and compared it with traditional ELISA.

Our findings reveal that immunoblot may be an alternative way for serologically diagnosing DH. Owing to the combination of tTG and npG on the b-aIB, reactions against both antigens can be detected simultaneously, thus widening, in a convenient way, the knowledge about the patient. Our results revealed a satisfactory level of agreement in anti-tTG IgA assessment (Cohen’s kappa value 0.78) in the b-aIB and ELISA.

In our selection of DH patients, we noticed certain discrepancies between DIF results and the results of serum examinations. Interestingly, according to the interpretation of Cohen’s kappa, there is a lack of interrater agreement between DIF and both ELISA and b-aIB. Intriguingly, based on literature reports, it is estimated that up to 10% of DH cases have a negative DIF reading [40,42]. Obviously, tests to confirm DH, including DIF, could be negative if a person was on a gluten-free diet for a long period of time. Probably, as was suggested by Sousa et al. [42], technical errors, failure of current laboratory methods in detecting cutaneous IgA deposits in some patients, and focal deposition of IgA in the skin may explain a negative DIF result in DH. Thus, considering that the failure to detect IgA is usually technical, DIF testing must be performed in experienced laboratories to minimize both false-positive and false-negative results [43].

Moreover, as we suggested previously [44], the proper biopsy site in DH patients is essential and determines the accuracy of the results (elimination of false-negative results). As was suggested by Zone et al. [45], a certain percent of skin biopsies from within lesions are negative because the inflammatory infiltrate destroys the antibody. We should also be aware that biopsy samples taken from the unaffected skin of the buttocks may be negative [44]. Thus, the decision about proper biopsy site should be taken individually in each patient.

Interestingly, as suggested by some authors [33,46], performing an aggressive gluten challenge after a gluten-free diet for at least one month can lead to a flare-up in lesion formation in about 24 h, thus confirming the diagnosis of DH in patients with negative DIF.

Some studies mention IgA subclasses in DH [15]. IgA1 is an IgA subclass that is often found in DH. IgA deposits in DH are polyclonal and mainly composed of IgA1, but the deposition of IgA2 suggests that they are, in part, of mucosal origin [38,47]. Therefore, perhaps the evaluation of IgA subclass deposits with DIF would reveal more positive results in our series of DH patients.

Generally speaking, the positive result of DIF is mandatory to diagnose DH; nevertheless, the real life-experience may not be so clear-cut. It is postulated that in the absence of the DIF characteristic pattern, the combination of clinical and immunologic data should support the DH recognition [42]. Thus, a negative Cohen’s kappa statistical result between serological tests and DIF may reflect a problem in the application of diagnostic tests. Our clinical-laboratory experience is that patients with lesions that clinically suggest DH and have a positive serological test result for IgA antibodies to tTG, but an initial negative reading of the traditional DIF specimen, cutting the DIF specimen further, and reexamining it for IgA DH-type deposits can yield a positive result. If it is still negative, then the biopsy for DIF should be repeated. This illustrates the spatial-temporal evolution of DH lesions at both clinical and microscopic levels that can be a limiting factor in diagnosing DH. It was also argued that the finding of IgA deposits in DH-compatible patterns using DIF may not necessarily mean that the diagnosis is indeed DH [48]. Moreover, according to our experience, the H&E histologic examination showing DH-suggestive features can skew the diagnosis toward DH, particularly if accompanied by serum studies showing DH-type autoimmune response.

It seems that anti-tTG IgA ELISA remains a simple and reliable diagnostic modality for DH; however, a large number of samples should be analyzed together in one test in order to optimize cost-effectiveness. On the other hand, with b-aIB a single sample may be analyzed without a negative economic impact on the diagnosis of DH. Both tests are automated immunoassays with objective assessment for circulating IgA against npG and tTG, but ELISA may be run in a quantitative format, which is useful in patient monitoring.

It is postulated that the age-dependent increase in anti-tTG IgA titer may be observed. Thus, the careful interpretation of immunoserological results, as well as the validity of the cutoff value, should be taken into account for rational therapeutic decisions in DH [49].

DH, CD, and IgA nephropathy share multiple nutritional and immunological factors. It is suggested that the overexpression of tTG may lead to the retrotranscythosis of IgA-bearing gliadin with increase IgA deposits. FcαRI/CD89 is probably involved in this mechanism by the direct interaction with gliadin and participation in the formation of IgA-sCD89 complexes [50]. Our previous studies [28,29] revealed the correlation between the intensity of CD89 cutaneous expression and anti-npG IgA detected with ELISA in DH, indicating that there is a single, shared pathway of DH-type IgA-mediated immune response to npG, tTG, and eTG.

Our findings are in line with the remarks of Salmi et al. [22] that circulating tTG antibodies support the diagnosis, but their absence does not exclude DH. However, in individuals who are negative for both DIF and anti-tTG, DH can be excluded [51]. While interpreting findings obtained by us, one should keep in mind the limitation of our work on DH, a relatively rare disease. We present the experiences of a single referral center, hence a relatively small number of DH patients. Our study design required that only active and untreated cases be selected. Still, this limitation did not preclude the appropriate statistical analysis enabling the interpretation of our data on DH-type IgA immune response.

## 5. Conclusions

Our conclusion is that both ELISA and b-aIB tests may be used for DH differential diagnosis and to indicate the intensity of gluten intolerance in DH. The ELISA diagnostics for anti-tTG IgA remains an optimal tool for serological analysis allowing quantitative assessment. The b-aIB may be used to support the diagnosis of DH. DIF remains a crucial laboratory test for DH identification; nevertheless, DH diagnosing requires careful consideration of all knowledge available about the given patient [52,53,54]. This is because clinical data, as well as the results of tissue imaging and serum biochemical-molecular techniques detecting DH-type IgA immune response, can be misleading when judged separately.

## Figures and Tables

**Table 1:** Characteristics of the examined groups.

Parameter	DH Group	Control Group (Healthy Subjects)
Number of patients	31	24
Sex	17 F; 14 M	15 F; 9 M
Mean age ± SD (min; max)	40.00 ± 19.65 (9; 80)	36.58 ± 10.12 (27; 65)
ELISA score (RU/mL)		
Anti-tTG IgA (mean ± SD)	134.99 ± 85.12	2.35 ± 1.58

Abbreviations: tTG—tissue transglutaminase, SD—standard deviation, min—minimum, max—maximum, F—female, M—male.

**Table 2:** The positive and negative results of ELISA and b-aIB in the subgroups of DH patients.

DH DIF-Positive Subgroup (n = 27)				DH DIF-Negative Subgroup (n = 4)	
Parameter		Results			
		Positive (n)	Negative (n)	Positive (n)	Negative (n)
ELISA	anti-tTG IgA	23	4	4	0
Bi-analyte immunoblot	anti-tTG IgA	17	10	4	0
	anti-npG IgA	23	4	4	0

**Table 3:** Calculation of the diagnostic sensitivity, specificity, and predictive values of bi-analyte immunoblot in relation to monoanalyte ELISA.

Parameters	Subjects (n)	Sensitivity (%)	Specificity (%)	PPV (%)	NPV (%)
tTG bi-analyte immunoblot vs. tTG ELISA	55	78	100	100	82
npG bi-analyte immunoblot vs. tTG ELISA	55	93	93	93	93
npG bi-analyte immunoblot vs. tTG bi-analyte immunoblot	55	100	82	78	100

Abbreviations: n—number of patients, tTG—tissue transglutaminase, npG—nonapeptides of gliadin, PPV—positive predictive value, NPV -negative predictive value.

**Table 4:** Interrater agreements (Cohen’s kappa values) among examined immunoserological systems for the antibodies tested.

Parameters	Cohen’s Kappa Values
tTG bi-analyte immunoblot vs. tTG ELISA	0.78
npG bi-analyte immunoblot vs. tTG ELISA	0.85
npG bi-analyte immunoblot vs. tTG bi-analyte immunoblot	0.78

Abbreviations: tTG—tissue transglutaminase, npG—nonapeptides of gliadin.

**Table 5:** Interrater agreements (Cohen’s kappa values) among examined immunoserological systems for the antibodies tested and DIF.

Parameters	Cohen’s Kappa Values
tTG bi-analyte immunoblot vs. DIF	−0.23
tTG ELISA vs. DIF	−0.15
npGbi-analyte immunoblot vs. DIF	−0.15

Abbreviations: DIF—direct immunofluorescence, tTG—tissue transglutaminase, npG—nonapeptides of gliadin.

## Data Availability

Patients’ documentation was archived at the Autoimmune Blistering Dermatoses Section of Department of Dermatology (Poznan University of Medical Sciences).

## References

[1] Szondy Z., Korponay-Szabó I., Kiraly R., Sarang Z., Tsay G.J. (2017). Transglutaminase 2 in human diseases. BioMedicine.

[2] Kárpáti S., Sárdy M., Németh K., Mayer B., Smyth N., Paulsson M., Traupe H. (2018). Transglutaminases in autoimmune and inherited skin diseases: The phenomena of epitope spreading and functional compensation. Exp. Dermatol..

[3] Lorand L., Iismaa S.E. (2019). Transglutaminase diseases: From biochemistry to the bedside. FASEB J..

[4] Liu C., Kellems R.E., Xia Y. (2017). Inflammation, Autoimmunity, and Hypertension: The Essential Role of Tissue Transglutaminase. Am. J. Hypertens..

[5] Ress K., Teesalu K., Annus T., Putnik U., Lepik K., Luts K., Uibo O., Uibo R. (2014). Low prevalence of IgA anti-transglutaminase 1, 2, and 3 autoantibodies in children with atopic dermatitis. BMC Res. Notes.

[6] Cascella N.G., Santora D., Gregory P., Kelly D.L., Fasano A., Eaton W.W. (2012). Increased Prevalence of Transglutaminase 6 Antibodies in Sera from Schizophrenia Patients. Schizophr. Bull..

[7] Teshima H., Kato M., Tatsukawa H., Hitomi K. (2020). Analysis of the expression of transglutaminases in the reconstructed human epidermis using a three-dimensional cell culture. Anal. Biochem..

[8] Gornowicz-Porowska J., Bowszyc-Dmochowska M., Dmochowski M. (2011). Autoimmunity-driven enzymatic remodeling of the dermal–epidermal junction in bullous pemphigoid and dermatitis herpetiformis. Autoimmunity.

[9] Antiga E., Maglie R., Quintarelli L., Verdelli A., Bonciani D., Bonciolini V., Caproni M. (2019). Dermatitis herpetiformis: Novel perspectives. Front. Immunol..

[10] Gornowicz-Porowska J., Bowszyc-Dmochowska M., Seraszek-Jaros A., Kaczmarek E., Dmochowski M. (2012). Association between Levels of IgA Antibodies to Tissue Transglutaminase and Gliadin-Related Nonapeptides in Dermatitis Herpetiformis. Sci. World J..

[11] Reunala T., Salmi T., Hervonen K. (2015). Dermatitis Herpetiformis: Pathognomonic Transglutaminase IgA Deposits in the Skin and Excellent Prognosis on a Gluten-free Diet. Acta Derm. Venereol..

[12] Bonciani D., Verdelli A., Bonciolini V., D’Errico A., Antiga E., Fabbri P., Caproni M. (2012). Dermatitis Herpetiformis: From the Genetics to the Development of Skin Lesions. Clin. Dev. Immunol..

[13] Koskinen L.L., Korponay-Szabo I.R., Viiri K., Juuti-Uusitalo K., Kaukinen K., Lindfors K., Mustalahti K., Kurppa K., Adány R., Pocsai Z. (2008). Myosin IXB gene region and gluten intolerance: Linkage to coeliac disease and a putative dermatitis herpetiformis association. J. Med. Genet..

[14] Duś M., Gornowicz J., Dmochowski M. (2009). Transient manifestation of dermatitis herpetiformis in a female with familial predisposition induced by propafenone. Postepy Dermatol. Alergol..

[15] Dmochowski M., Gornowicz-Porowska J., Bowszyc-Dmochowska M. (2019). An update on direct immunofluorescence for diagnosing dermatitis herpetiformis. Adv. Dermatol. Allergol..

[16] Bresler S.C., Granter S.R. (2015). Utility of Direct Immunofluorescence Testing for IgA in Patients with High and Low Clinical Suspicion for Dermatitis Herpetiformis. Am. J. Clin. Pathol..

[17] Hull C., Liddle M., Hansen N., Meyer L., Schmidt L., Taylor T., Jaskowski T., Hill H., Zone J. (2008). Elevation of IgA anti-epidermal transglutaminase antibodies in dermatitis herpetiformis. Br. J. Dermatol..

[18] Valencia-Guerrero A., Dresser K., Cornejo K.M. (2018). The utility of tissue and epidermal transglutaminase immunohistochemistry in dermatitis herpetiformis. Indian J. Dermatopath Diagn Dermatol..

[19] Kumar V., Jarzabek-Chorzelska M., Sulej J., Rajadhyaksha M., Jablonska S. (2001). Tissue transglutaminase and endomysial antibodies-diagnostic markers of gluten-sensitive enteropathy in dermatitis herpetiformis. Clin. Immunol..

[20] Sankari H., Hietikko M., Kurppa K., Kaukinen K., Mansikka E., Huhtala H., Laurila K., Reunala T., Hervonen K., Salmi T. (2020). Intestinal TG3- and TG2-Specific Plasma Cell Responses in Dermatitis Herpetiformis Patients Undergoing a Gluten Challenge. Nutrients.

[21] Rose C., Dieterich W., Bröcker E.-B., Schuppan D., Zillikens D. (1999). Circulating autoantibodies to tissue transglutaminase differentiate patients with dermatitis herpetiformis from those with linear IgA disease. J. Am. Acad. Dermatol..

[22] Salmi T.T. (2019). Dermatitis herpetiformis. Clin. Exp. Dermatol..

[23] Kasperkiewicz M., Dähnrich C., Probst C., Komorowski L., Stöcker W., Schlumberger W., Zillikens D., Rose C. (2012). Novel assay for detecting celiac disease-associated autoantibodies in dermatitis herpetiformis using deamidated gliadin-analogous fusion peptides. J. Am. Acad. Dermatol..

[24] GGornowicz-Porowska J., Seraszek-Jaros A., Bowszyc-Dmochowska M., Kaczmarek E., Pietkiewicz P., Bartkiewicz P., Dmochowski M. (2017). Accuracy of molecular diagnostics in pemphigus and bullous pemphigoid: Comparison of commercial and modified mosaic indirect immunofluorescence tests as well as enzyme-linked immunosorbent assays. Adv. Dermatol. Allergol..

[25] Harrell J., Rubio X.B., Nielson C., Hsu S., Motaparthi K. (2019). Advances in the diagnosis of autoimmune bullous dermatoses. Clin. Dermatol..

[26] Jones A.L., Dhanapala L., Kankanamage R.N.T., Kumar C.V., Rusling J.F. (2020). Multiplexed Immunosensors and Immunoarrays. Anal. Chem..

[27] Heyman M., Ménard S. (2009). Pathways of Gliadin Transport in Celiac Disease. Ann. N. Y. Acad. Sci..

[28] Gornowicz-Porowska J., Seraszek-Jaros A., Bowszyc-Dmochowska M., Kaczmarek E., Dmochowski M. (2018). Immunoexpression of IgA receptors (CD89, CD71) in dermatitis herpetiformis. Folia Histochem. Cytobiol..

[29] Gornowicz-Porowska J., Seraszek-Jaros A., Bowszyc-Dmochowska M., Kaczmarek E., Pietkiewicz P., Bartkiewicz P., Dmochowski M. (2017). A comparative study of expression of Fc receptors in relation to the autoantibody-mediated immune response and neutrophil elastase expression in autoimmune blistering dermatoses. Pol. J. Pathol..

[30] Bonciolini V., Bonciani D., Verdelli A., D’Errico A., Antiga E., Fabbri P., Caproni M. (2012). Newly described clinical and immunopathological feature of dermatitis herpetiformis. Clin. Dev. Immunol..

[31] Antiga E., Bonciolini V., Cazzaniga S., Alaibac M., Calabrò A.S., Cardinali C., Cozzani E., Marzano A.V., Micali G., Not T. (2019). Female Patients with Dermatitis Herpetiformis Show a Reduced Diagnostic Delay and Have Higher Sensitivity Rates at Autoantibody Testing for Celiac Disease. BioMed Res. Int..

[32] Gasparini G., Cozzani E., Caproni M., Antiga E., Signori A., Parodi A. (2019). Could anti-glycan antibodies be useful in dermatitis herpetiformis?. Eur. J. Dermatol..

[33] Beutner E.H., Baughman R.D., Austin B.M., Plunkett R.W., Binder W.L. (2000). A case of dermatitis herpetiformis with IgA endomysial antibodies but negative direct immunofluorescence findings. J. Am. Acad. Dermatol..

[34] Reunala T., Salmi T.T., Hervonen K., Kaukinen K., Collin P. (2018). Dermatitis Herpetiformis: A Common Extraintestinal Manifestation of Coeliac Disease. Nutrients.

[35] Mansikka E., Salmi T., Kaukinen K., Collin P., Huhtala H., Reunala T., Hervonen K. (2018). Diagnostic Delay in Dermatitis Herpetiformis in a High-prevalence Area. Acta Derm. Venereol..

[36] Ziberna F., Sblattero D., Lega S., Stefani C., Dal Ferro M., Marano F., Gaita B., De Leo L., Vatta S., Berti I. (2021). A novel quantitative ELISA as accurate and reproducible tool to detect epidermal transglutaminase antibodies in patients with Dermatitis Herpetiformis. J. Eur. Acad. Dermatol. Venereol..

[37] Marietta E.V., Camilleri M.J., Castro L.A., Krause P.K., Pittelkow M.R., Murray J.A. (2008). Transglutaminase Autoantibodies in Dermatitis Herpetiformis and Celiac Sprue. J. Investig. Dermatol..

[38] Dieterich W., Schuppan D., Laag E., Bruckner-Tuderman L., Reunala T., Kárpáti S., Zágoni T., Riecken E.O. (1999). Antibodies to Tissue Transglutaminase as Serologic Markers in Patients with Dermatitis Herpetiformis. J. Investig. Dermatol..

[39] Clarindo M.V., Possebon A.T., Soligo E.M., Uyeda H., Ruaro R.T., Empinotti J.C. (2014). Dermatitis herpetiformis: Pathophysiology, clinical presentation, diagnosis and treatment. An. Bras. Dermatol..

[40] Desai A.M., Krishnan R.S., Hsu S. (2005). Medical Pearl: Using tissue transglutaminase antibodies to diagnose dermatitis herpetiformis. J. Am. Acad. Dermatol..

[41] Mendes F.B.R., Hissa-Elian A., De Abreu M.A.M.M., Gonçalves V.S. (2013). Review: Dermatitis herpetiformis. An. Bras. Dermatol..

[42] Sousa L., Bajanca R., Cabral J., Fiadeiro T. (2002). Dematitis herpetiformis: Should direct immunofluorescence be the only diagnostic criterion?. Pediatr. Dermatol..

[43] Caproni M., Antiga E., Melani L., Fabbri P., The Italian Group for Cutaneous Immunopathology (2009). Guidelines for the diagnosis and treatment of dermatitis herpetiformis. J. Eur. Acad. Dermatol. Venereol..

[44] Lutkowska A., Pietkiewicz P., Sulczyńska-Gabor J., Gornowicz J., Bowszyc-Dmochowska M., Dmochowski M. (2009). Gluteal skin is not an optimal biopsy site for direct immunofluorescence in diagnostics of dermatitis herpetiformis. A report of a case of dermatitis herpetiformis Cottini. Dermatol. Klin..

[45] Zone J.J., Meyer L.J., Petersen M.J. (1996). Deposition of granular IgA relative to clinical lesions in dermatitis herpetiformis. Arch. Dermatol..

[46] Beutner E.H., Plunkett R.W. (2006). Methods for diagnosing dermatitis herpetiformis. J. Am. Acad. Dermatol..

[47] Wojnarowska F., Delacroix D., Gengoux P. (1988). Cutaneous IgA subclasses in dermatitis herpetiformis and linear IgA disease. J. Cutan. Pathol..

[48] Antiga E., Maglie R., Lami G., Tozzi A., Bonciolini V., Calella F., Bianchi B., Bianco E., Renzi D., Mazzarese E. (2021). Granular Deposits of IgA in the Skin of Coeliac Patients without Dermatitis Herpetiformis: A Prospective Multicentric Analysis. Acta Derm. Venereol..

[49] Nakazawa H., Makishima H., Ito T., Ota H., Momose K., Sekiguchi N., Yoshizawa K., Akamatsu T., Ishida F. (2014). Screening Tests Using Serum Tissue Transglutaminase IgA May Facilitate the Identification of Undiagnosed Celiac Disease among Japanese Population. Int. J. Med. Sci..

[50] Lerner A., Berthelot L., Jeremias P., Matthias T., Abbad L., Monteiro R.C. (2017). Gluten, Transglutaminase, Celiac Disease and IgA Nephropathy. J. Clin. Cell. Immunol..

[51] Antiga E., Caproni M. (2015). The diagnosis and treatment of dermatitis herpetiformis. Clin. Cosmet. Investig. Dermatol..

[52] Skręta-Śliwińska M., Woźniacka A., Żebrowska A. (2020). Haemorrhagic form of dermatitis herpetiformis. Dermatol. Rev..

[53] Verdelli A., Corrà A., Caproni M. (2021). Reply letter to “An update on direct immunofluorescence for diagnosing dermatitis herpetiformis”. Could granular C3 deposits at the dermal epidermal junction be considered a marker of “cutaneous gluten sensitivity”?. Postępy Dermatol. Alergol..

[54] Görög A., Antiga E., Caproni M., Cianchini G., De D., Dmochowski M., Dolinsek J., Drenovska K., Feliciani C., Hervonen K. (2021). S2k guidelines (consensus statement) for diagnosis and therapy of dermatitis herpetiformis initiated by the European Academy of Dermatology and Venereology (EADV). J. Eur. Acad. Dermatol. Venereol..

